# Investigating the Effectiveness of Nano-Montmorillonite on Asphalt Binder from Rheological, Thermodynamics, and Chemical Perspectives

**DOI:** 10.3390/ma14061433

**Published:** 2021-03-16

**Authors:** Peifeng Cheng, Zhanming Zhang, Zonghao Yang, Jin Xu, Yiming Li

**Affiliations:** 1 Civil Engineering Department, Northeast Forestry University, Harbin 150040, China; chengpeifeng@nefu.edu.cn (P.C.); nefusonny@126.com (Z.Y.); li_yiming2017@163.com (Y.L.); 2 Key Laboratory of Road Structure and Green Ecological Technology in Heilongjiang Province, Northeast Forestry University, Harbin 150040, China; 3 Heilongjiang Longer Engineering Design Co., Ltd., Harbin 150040, China; hrbxujin@126.com

**Keywords:** road engineering, asphalt binder, nano-montmorillonite, rheological property, thermodynamics, chemical structure

## Abstract

In this research, the feasibility of using nano-montmorillonite (MMT) in asphalt binders was investigated in terms of rheological properties, thermomechanical properties, and chemical structure composition. Different doses of MMT were added to the base asphalt and styrene–butadiene–styrene (SBS) asphalt as test subjects. The effect of nanomaterials on the high-temperature resistance of asphalt binders to permanent deformation was analyzed from dynamic mechanical rheology using the multiple stress creep recovery (MSCR) test. The sessile drop method test based on surface free energy (SFE) theory was employed and thermodynamic parameters such as surface free energy, cohesive work, and adhesion work were calculated to analysis the change in energy of the asphalt binder. In addition, changes in the chemical structure and composition of the asphalt binder were examined by Fourier transform infrared (FTIR) and gel permeation chromatography (GPC) tests. The results showed that MMT can effectively enhance the high-temperature elastic recovery and plastic deformation resistance of the asphalt binder. The intercalation structure produced in the asphalt binder enhanced the overall cohesive power and adhesion to the aggregate. The anchoring effect of the intercalation structure resulted in an increase in the macromolecular weight of the binder was demonstrated, indicating that MMT enhanced the overall intermolecular forces of the binder. In addition, the molecular crystal structure was characterized by characteristic functional groups in the infrared spectra, while demonstrating that no chemical reaction occurs during the modification of the binder by the nanomaterials.

## 1. Introduction

Asphalt is a common pavement material in various countries around the world, but it often faces some problems in service, such as high sensitivity to temperature, softening under high-temperature conditions, cracking under low-temperature conditions, and aging under high-temperature and ultraviolet light [1]. In order to improve the road performance of asphalt pavement, the addition of modifier to the base asphalt has become a development trend. The most-used modifiers with significant effects are the polymer modifier; polymer modifier can significantly improve the low-temperature cracking performance and high-temperature stability of asphalt materials, including styrene–butadiene–styrene (SBS) block copolymer for the most widely used polymer modified materials [2]. However, in the application of road projects, the base asphalt and SBS modified asphalt have a certain degree of durability, but with the increase in service life, under the effects of natural environmental factors, asphalt gradually hardens, becomes brittle, cracks, and cannot continue to perform the original adhesion and sealing function, resulting in poor performance [3,4]. Pavement in the actual use of the process is also subject to alternating stress, cyclic stress, and the comprehensive effect of the external environment, and then produces serious disease, and the actual road performance and service life of asphalt concrete pavement impact, ultimately leading to a decline in pavement quality and affecting the service function of the road.

With the rapid development of nanotechnology, the processing of nanomaterials has become easier, and their industrial production scale has increased rapidly. The nanoscale size gives nanomaterials special physical and chemical properties that are different from other materials, and these advantages are of interest to road researchers. Nanotechnology is gradually appearing in the field of pavement engineering, especially in the application of nanomaterials as modifiers to asphalt binder. Different nanomaterials optimized the properties of asphalt binders have been investigated by scholars in recent studies. Li et al. [5] demonstrated that the addition of graphene nanoparticles to asphalt can effectively improve the aging resistance, high-temperature stability, and temperature sensitivity of asphalt by studying the performance of asphalt modified with different amounts of graphene. Graphene was homogeneously dispersed as asphaltene with high surface energy and acted as a micellar nucleus in the bitumen. The increased viscosity and graphene flakes hinder and prevent oxygen diffusion into the bitumen. The results of the study by Bhat et al. [6] show that the addition of nano-Al_2_O_3_ has a positive effect on the rutting and fatigue properties of SBS modified bitumen binders. The storage stability of the binder was significantly improved with the addition of nano-Al_2_O_3_, and its aging resistance was also improved. Wang et al. [7] investigated the feasibility of incorporating graphene oxide and carbon nanotubes into SBS modified bitumen separately. Wu et al. [8] used uniaxial static compression creep dynamic shear rheological (DSR) tests and dynamic modulus tests on asphalt mixtures to demonstrate that the nano-TiO_2_/CaCO_3_ composite modified asphalt has. The nano-TiO_2_/CaCO_3_ composite modified asphalt has good rutting resistance at high temperatures and has improved cracking resistance at low temperatures. The different dimensions of the nanomaterials have been shown to be feasible for use in road engineering due to their characteristics.

Among them, MMT as a two-dimensional nanomaterial has been widely studied by scholars because of its ability to modify many properties of asphalt [9,10,11]. Nano-montmorillonite is a type of layered silicate with a thickness of less than 10 nm and therefore has a very large value of aspect ratio. With a specific surface area of 700–800 m^2^/g, it has excellent surface activity, interacts with polymers and forms a dense structure. The physical properties of asphalt, such as stiffness, tensile strength, flexural strength, tensile modulus, and thermal stability, can be significantly improved by adding a small amount of MMT to the asphalt. The addition of a small amount of nano-montmorillonite to asphalt can cause the physical properties of asphalt to be significantly improved, such as stiffness, tensile strength, flexural strength, tensile modulus, and thermal stability [12,13,14]. It shows a peeling type of dispersion in asphalt, and due to its large layer gap, it can absorb light asphalt components such as saturated hydrocarbons and aromatics, thus improving the high-temperature properties of asphalt [15,16]. The surface of the polymer particles is adhered by MMT, which leads to a reduction in the density difference between the polymer and the asphalt, and therefore the polymer-modified asphalt has good stability [17,18]. The lamellar edges of the montmorillonite modified by quaternary organic ammonium salts are more regular and without significant curling. Asphalt molecules can undergo exchange reactions with cations and insert into the lamellar spaces of organic montmorillonite, improving the high-temperature properties and storage stability of the asphalt but reducing the low-temperature properties [19].

The addition of MMT was shown to improve the fatigue life of HMA by conventional fatigue life and dissipation energy evaluation indexes. The results of surface free energy measurements also showed that the increase of nano-additives led to changes in the acidic and alkaline components of the modified asphalt [20]. The best resistance to permanent deformation of SMA asphalt mixes with 3% MMT addition was verified by Hamburg wheel diameter test and dynamic creep test [21]. Multiple damage-healing tests of asphalt mixtures accompanied by intermittent time were conducted by semicircular bending test, and the results showed that MMT can effectively enhance the healing performance of asphalt mixtures with microcracks [22]. MMT and SBS showed significant improvement in the rutting resistance of modified steel slag asphalt mixtures, and the nanomaterials also improved the toughness and viscosity of asphalt by different magnitudes [23]. In addition, the lamellar structure of organic montmorillonite greatly impedes the movement of oxygen radicals in the asphalt, resulting in good aging resistance of the base asphalt or polymer modified asphalt binders [24,25,26]. As a result, MMT can have investigable value and positive impact by potentially improving one or some properties of asphalt binder and mixture.

In conclusion, MMT has been chosen as a modifier for asphalt with a number of distinct advantages. Base asphalt and SBS asphalt binder were chosen as the two most commonly used materials for roads today, and organically treated nano-montmorillonite was added to the asphalt at different dosing levels. First, the multiple stress creep recovery test was employed for rheological index correlation analysis. Subsequently, the sessile drop test was applied to test and analyze the thermodynamic parameters by surface energy theory. In addition, gel permeation chromatography test and infrared spectroscopy test were employed as chemical fields to analyze the changes in molecular weight distribution and characteristic functional groups as indicators. The effects of MMT on asphalt properties from rheological, thermodynamic, and chemical perspectives were investigated and analyzed in this research.

## 2. Materials and Methods

### 2.1. Materials

AH 90 asphalt was obtained from Panjin, Liaoning Province, and SBS modified asphalt (4.5 wt% SBS) was provided by a chemical plant in Shandong Province; the basic physical properties are shown in Table 1. The modifier was provided by a Guangdong chemical company with octadecyl dimethyl benzyl ammonium chloride organically treated sodium-based nano-montmorillonite, whose excellent lipophilic properties make it easier to bond with asphalt binder, the properties of which is shown in Table 2. Three aggregates commonly used in road construction were collected on road projects under construction in Heilongjiang Province, and their main chemical compositions are shown in Table 3.

### 2.2. Sample Preparation

Modified asphalt was prepared continuously at high temperature with mechanical agitator and high-speed magnetic shear machine. The preparation process parameters have been optimized and determined in previous research [22]. Firstly, the base asphalt and SBS modified asphalt binder were heated to the melting state of 160 °C and 180 °C respectively in an insulated stainless-steel vessel, and then the MMT with design content (1 wt%, 3 wt%, and 5 wt%) was added to the asphalt and stirred at low speed for 30 min. Then, a high-speed shear machine was used to shear for 30 min at 5000 rpm. Finally, it was stirred again at a low speed for 30 min to ensure that the modifier was fully integrated into the asphalt. The names of asphalt binder samples are shown in Table 4.

**Figure 1:**
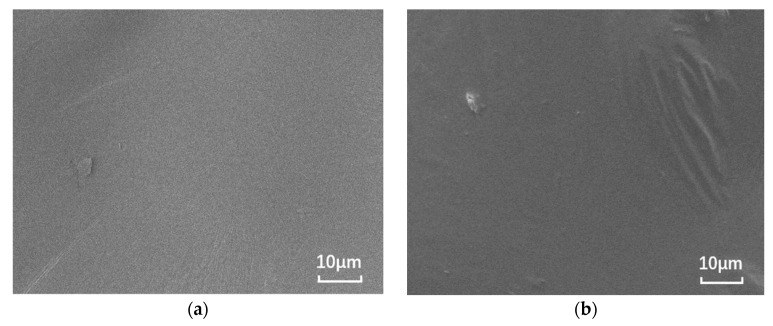

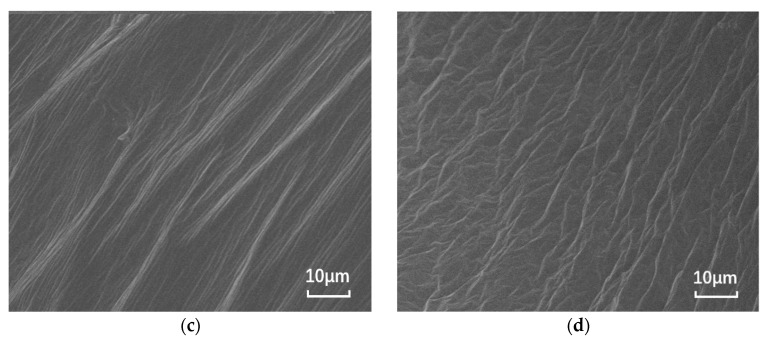
SEM images of different asphalt binders: (**a**) B0; (**b**) S0; (**c**) B5; and (**d**) S5.

The nano-montmorillonite was added to the asphalt and prepared, and significant microscopic differences are observed in Figure 1. The SEM images of the base asphalt and SBS modified asphalt binder are smooth, while the surfaces of the samples with added MMT show “branches” and “waves”, respectively. This is due to the irregular shape caused by the MMT forming intercalated structures in the binder and the MMT adsorbing onto the SBS due to the expansion between the SBS and asphalt phases.

### 2.3. Multiple Stress Creep Recovery Test

In order to investigate the effect of MMT on the ability of asphalt binder to resist permanent deformation at high temperature, a multiple stress creep recovery test (MSCR) was performed on asphalt binder using an Anton Paar MCR-302 advanced rotational rheometer. Asphalt binder samples were made into a disk shape through the use of a silicone mold. The diameter and thickness were 25 and 1 mm. A test temperature of 60 °C was chosen as the test temperature, and the asphalt sample to be tested was subjected to 10 cycles at 0.1 kPa stress, where each cycle underwent 1 s of creep and 9 s of recovery deformation. This was followed by 10 successive cycles of creep recovery at 3.2 kPa stress. The test results of asphalt samples were automatically collected during the test, and the creep recovery rate (R) and nonrecoverable creep flexibility (Jnr) of asphalt were calculated based on the strain information to evaluate the elastic recovery performance of modified asphalt at specific temperature.

### 2.4. Sessile Drop Method Test

The Dataphysics OCA-20 optical contact angle meter was used to test the contact angle of the asphalt binder. Compared with the Wilhelmy plate approach, the solid drop method requires lower test conditions and is universal [27,28]. The tests were performed at dry room temperature. The test liquids were selected as deionized water, glycerin, and formamide. In addition, the surface energy parameters of the aggregate were measured in order to determine the adhesion work between the asphalt binder and the aggregate. Limestone, sandstone, and basalt aggregate samples were cut into rectangles (20 mm × 20 mm × 10 mm) using a small cutter and polished with sandpaper on the surface to be measured. Each probe drop was placed at five different locations on the sample surface, the test results were recorded, and dispersive component of surface energy ($\gamma^{d}$), polar component of surface energy ($\gamma^{p}$), and surface free energy (γ) of test liquid and aggregate were calculated as shown in Table 5.

### 2.5. Fourier Transform Infrared Spectroscopy Test

A Thermo Fisher Nicolet iS 50 FTIR spectrometer was used to test the characteristic functional groups and chemical properties of the asphalt binder. The resolution was 0.09 cm^−1^ and the number of scans was 60. The asphalt binder was prepared in a 1:19 solution with carbon disulfide solution and allowed to be dropped on a potassium bromide window pan, spread evenly and allowed to dry completely before the sample was tested. The scan range for the test was selected to be 4000 cm^−1^ to 400 cm^−1^.

### 2.6. Gel Permeation Chromatography Test

The molecular weight distribution of the asphalt binder was tested using an Agilent 1100 liquid chromatograph. Approximately 20 mg of asphalt binder sample was dissolved in 10 mL of tetrahydrofuran (THF). The GPC spectra were partitioned into three intervals and 13 sections using Origin’s peak analyzer. The three intervals were large molecular size (LMS, corresponding to sections 1–5), medium molecular size (MMS, corresponding to sections 6–9), and small molecular size (SMS, corresponding to sections 10–13) [29,30]. In addition, the weight-averaged molecular weight (Mw), number-averaged molecular weight (Mn), and dispersion (D) were also calculated in this paper.

## 3. Results

### 3.1. Rheological Performance Analysis

#### 3.1.1. Termination Strain of MSCR Test

**Figure 2:**
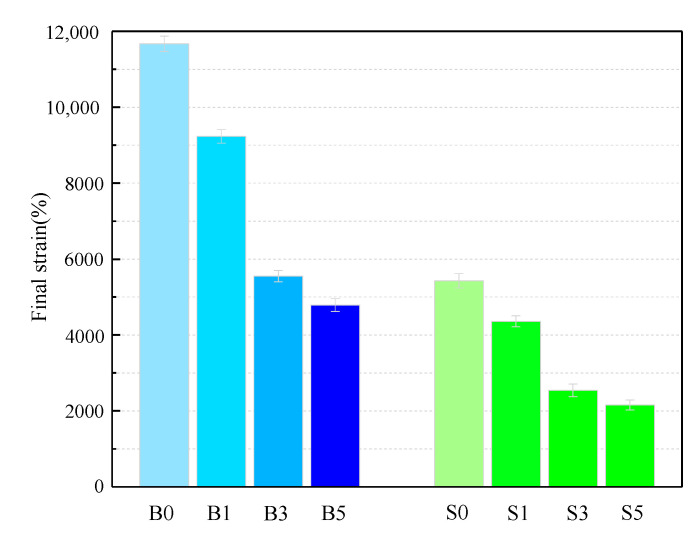
Final strain of asphalt binder samples.

Considered in terms of resistance to plastic deformation, good high-temperature performance of asphalt binder can be interpreted as a small plastic deformation of the binder under the same loading conditions. Alternatively, under the same conditions and strains, the binder has a high strain recovery capacity. The magnitude of the likelihood of rutting in asphalt pavements under high-temperature action is reflected by these two points. After the MSCR test at 60 °C, the final strain of samples is shown in Figure 2 and Table 6. The total strain of the S0 sample was 46.50% of that of the B0 sample, which means that the SBS asphalt binder has a better elastic recovery than the base asphalt and that it has a better resistance to plastic deformation. The trend in the figure shows that the overall plastic deformation of the asphalt binder decreases significantly with the increase in MMT content, with the strain values of the B3 and S3 samples showing a significant decrease. Even the strains of B5 and S5 decreased by 49.03% and 46.12%. Thus, MMT significantly improves the ability of the asphalt binder to resist plastic deformation at 60 °C.

#### 3.1.2. Deformation Recovery Percentage (R%)

This parameter represents the creep recovery rate of the asphalt binder after being applied a load. The calculated results of the R parameter are complementary to those of the Jnr parameter, and the asphalt viscoelastic properties can be more completely represented by combining their experimental results. The value of R and its difference ratio of the parameters are calculated in Equations (1) and (2).

(1) $$R=\frac{\varepsilon_{p}-\varepsilon_{r}}{\varepsilon_{p}-\varepsilon_{0}}\times 100$$

(2) $$R_{diff}=\frac{R_{0.1}-R_{3.2}}{R_{0.1}}\times 100$$

where R is the deformation recovery percentage, %; $R_{diff}$ is the creep recovery rate difference ratio, %; $\varepsilon_{p}$ is the peak strain in each loading cycle, %; $\varepsilon_{r}$ is the residual strain in each loading cycle, %; and $\varepsilon_{0}$ is the initial strain in each loading cycle, %.

**Figure 3:**
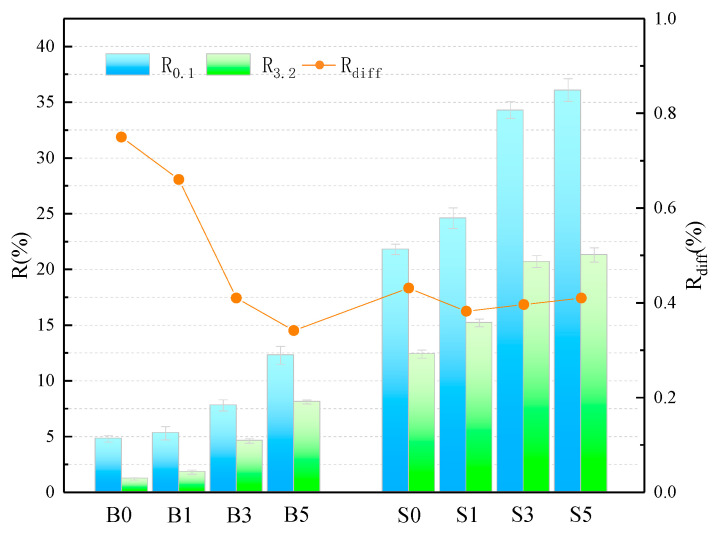
Calculation of the relevant parameters for the R.

The average creep recovery and creep recovery difference ratios of the samples at 0.1 kPa and 3.2 kPa stress levels are shown in Figure 3 and Table 6. The smallest value of elastic recovery was found for the B0 sample, with an R-value of only 1.2% at 3.2 kPa, indicating the poor elastic recovery of the base asphalt at 60 °C. The SBS asphalt binder has a high R-value, which proves its superior performance in terms of elastic recovery. The SBS asphalt binder, on the other hand, possesses a high R-value, which also proves that its superior high-temperature performance contributes partially to the elastic recovery ability. In addition, it is evident that the R-value of the asphalt binder obtained a gradual increase with the increase of MMT doping. When the MMT admixture was 5 wt%, the R-values increased by 256.25% and 149.05% in the base asphalt and SBS asphalt binder, respectively, at a loading condition of 0.1 kPa. This fully illustrates that MMT has a significant contribution to the high-temperature elastic recovery of asphalt, and its intercalation structure plays a role in coordinating the force and deformation of the binder system. In addition, the larger the $R_{diff}$, the higher the sensitivity of the asphalt elastic recovery capacity to the heavy load effect. As can be seen from the figure, the elastic recovery rate differential ratio of the base asphalt is the largest, reaching 75%, while the $R_{diff}$ of the asphalt decreases gradually with the addition of MMT, and even the B5 sample has been lower than the S0 sample. This indicates that in higher temperature environment, MMT reduces the sensitivity of asphalt creep recovery ability for heavy load action. Moreover, the addition of MMT to the SBB asphalt binder caused a slight decrease in the value of $R_{diff}$, implying a slight decrease in its elastic recovery capacity.

#### 3.1.3. Nonrecoverable Creep Compliance (J_nr_)

Jnr is the ratio of plastic strain to stress in asphalt during the creep cycle. The larger the Jnr under the same conditions, the lower the binder’s resistance to plastic deformation, meaning that a higher-temperature performance is worse; conversely, the higher-temperature performance of the binder is better. *J_nr_* and $J_{diff}$ are calculated as in Equations (3) and (4).

(3) $$J_{nr}=\frac{\varepsilon_{r}-\varepsilon_{0}}{\sigma }$$

(4) $$J_{diff}=\frac{J_{0.1}-J_{3.2}}{J_{3.2}}\times 100$$

where, *J_nr_* is nonrecoverable creep compliance, Pa^−1^; $J_{diff}=\frac{J_{0.1}-J_{3.2}}{J_{3.2}}\times 100$ is the difference ratio of nonrecoverable creep compliance, %; and *σ* is the applied stress level, Pa.

**Figure 4:**
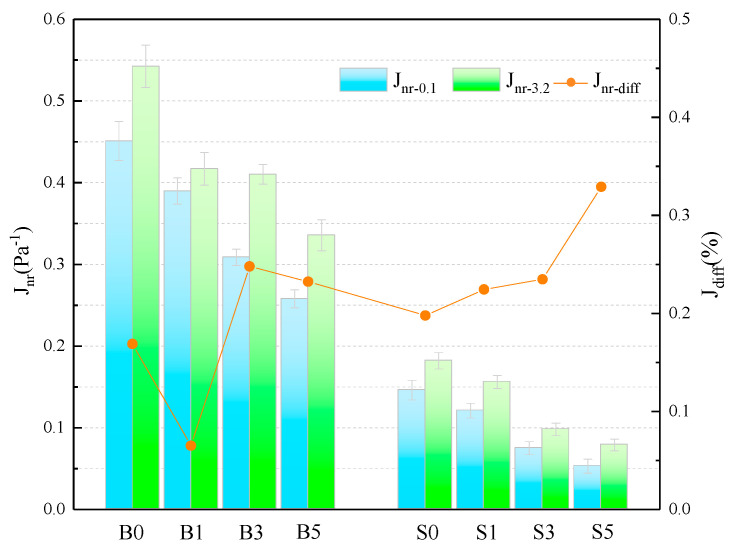
Calculation of the relevant parameters for the *J_nr_*.

The *Jnr* and $\;J_{diff}$ of different samples are presented in Figure 4 and Table 6. The *J_nr_* of SBS asphalt binder is only 32.38% and 33.56% of that of the base asphalt at 0.1 kPa and 3.2 kPa stress levels and 60 °C. Adding MMT to asphalt binder, the Jnr of binder is decreasing as the admixture level increases. This shows that MMT can improve the resistance of asphalt to plasticity degeneration at high temperature, which in turn improves the high-temperature performance of binder. The magnitude of the $J_{diff}$ value characterizes the sensitivity of the Jnr of the asphalt binder to the action of heavy loads. Its larger value means that the binder is more sensitive to heavy load traffic and its creep recovery ability is poor [31]. The calculated results show that although the values of some groups do not show a consistent trend, it is undeniable that the values of $J_{diff}$ increase to varying degrees when the amount of MMT is increased. This indicates that the addition of MMT, especially in larger amounts, increases the sensitivity of Jnr of asphalt binder to heavy loads and reduces the creep recovery capacity.

### 3.2. Surface Free Energy Analysis

#### 3.2.1. Surface Free Energy and Its Components

The adhesion of asphalt binder and aggregate is the main factor affecting the service performance of asphalt pavement, and the adhesion performance of the two-phase system can be studied by applying surface free energy. Therefore, in recent years, surface free energy theory has been widely used to analyze the adhesion properties of these two materials. The increase in Gibbs free energy per unit in specific surface area when keeping temperature, pressure and composition constant is defined as the surface free energy. In asphalt mixture, the adhesion between binder and aggregate is due to the wetting of the aggregate surface by asphalt, and this process can be considered as a reduction in the surface free energy of the asphalt-aggregate composite system. According to research of Fowkes, Good, and Van [32,33,34], the surface tension of solids and liquids consists of dispersive and polar components; therefore, the surface free energy can be calculated by the following equation.

(5) $$\gamma =\gamma^{d}+\gamma^{p}$$

where γ is the surface free energy, mJ/m^2^; $\gamma^{d}$ is the dispersive component of surface energy, mJ/m^2^; and $\gamma^{p}$ is the polarity component of surface energy, mJ/m^2^.

Fowkes demonstrated that the dispersion force between liquid and solid can be expressed as the liquid–solid surface free energy dispersion component [31]. Owens developed this approach and made it applicable to polar components [35]. Thus, for solid–liquid interface, the surface free energy is calculated as in Equation (6).

(6) $$\gamma_{\mathrm{SL}}=\gamma_{S}+\gamma_{L}-2\sqrt{\gamma_{S}^{d}\gamma_{L}^{d}}-2\sqrt{\gamma_{S}^{p}\gamma_{L}^{p}}$$

where $\gamma_{\mathrm{SL}}$ is the surface free energy at the solid–liquid interface, mJ/m^2^; $\gamma_{S}$ is the surface free energy of the solid, mJ/m^2^; $\gamma_{L}$ is the surface free energy of the liquid, mJ/m^2^; $\gamma_{S}^{d}$ is the dispersion component of the surface free energy of a solid, mJ/m^2^;${\;\gamma }_{L}^{d}$ is the dispersion component of the surface free energy of the liquid, mJ/m^2^;${\;\gamma }_{S}^{p}$ is the polar component of the surface free energy of a solid mJ/m^2^; and $\gamma_{L}^{p}$ is the polar component of liquid surface free energy, mJ/m^2^.

Young’s equation as in Equation (7) [36].

(7) $$\gamma_{S}=\gamma_{\mathrm{SL}}+\gamma_{L}cos\theta$$

where *θ* is the contact angle, °. Combined with Equation (7), Equation (8) can be obtained.

(8) $$\gamma_{L}1+cos\theta =2\sqrt{\gamma_{S}^{d}\gamma_{L}^{d}}+2\sqrt{\gamma_{S}^{p}\gamma_{L}^{p}}$$

In Equation (8), $\gamma_{S}^{d}$ and $\gamma_{S}^{p}$ are unknowns, while the other terms are known, and the surface energy parameters of another solid to be measured can be obtained by measuring the contact angle using two liquids with known surface energy parameters. The surface energy parameter of the solid $\gamma_{S}$ to be measured can be obtained from Equation (5).

The surface energy parameters of base asphalt and SBS asphalt binder mixed with different levels of MMT is demonstrated in Table 7. It can be seen that the SFE values of base asphalt and SBS asphalt binder are increased by 54% and 20.7%, respectively, for dosages ranging from 0 to 5%. Therefore, we found that MMT can increase the total SFE of asphalt binder. The adhesion between the binder and the aggregate is formed by the asphalt infiltrating the surface of the aggregate, and the process of this infiltration is a reduction of the surface free energy of the solid–liquid composite system. The higher the surface free energy, the better adhesive attraction between asphalt and aggregate. In addition, it can also be noticed that the polar component of surface energy does not change significantly with the increase of MMT modifier dosage. However, the dispersion component gradually increases, which is because asphalt is a non-polar material and its polar component occupies a small proportion. The larger the value of the dispersion component, the stronger the adhesion performance of the asphalt. The addition of MMT to the asphalt binder causes the intercalation structure to be formed, especially in SBS-modified asphalt, where the SBS molecular structure dispersed in the asphalt forms a physical connection system with the lamellar structure of MMT. The overall polarity of the system is enhanced by the combined effect of intermolecular forces and the linking of chemical bonds by hydrogen bonding due to the electron movement between asphaltenes.

#### 3.2.2. Cohesion and Adhesion Work

There are two possible locations in asphaltic materials that can cause cracking, through the interior of the asphalt binder and along the interface between the binder and the aggregate. The energy required to resist crack propagation in binder is called cohesive work and is twice as much as the material SFE. The relationship is shown in Equation (9).

(9) $${\Delta G}_{\mathrm{coh}}=2\gamma_{\mathrm{binder}}^{d}+2\gamma_{\mathrm{binder}}^{p}$$

The effect of MMT on the cohesive work and adhesion work is shown in Table 7. According to the introduction of Equation (9), the numerical magnitude of the cohesive work is two times the value of the surface free energy, so both have the same trend of change. By comparison, it is found that the cohesive work of SBS-modified asphalt is higher than that of base asphalt, and the enhancement effect of MMT on the cohesive work of base asphalt is better than that of SBS modified asphalt binder, and the cohesive work can be enhanced by 51.8% and 20.7%, respectively, compared with asphalt without added nanomaterials. It is worth mentioning that the cohesive power of 5% MMT added to the base asphalt has exceeded that of SBS asphalt binder. Therefore, according to the calculation results, it can be seen that the addition of MMT makes the asphalt exhibit better cohesion and helps to enhance the resistance of asphalt to cohesive damage. The reason may be that the better dispersion of MMT increases the cohesive power of the base asphalt, and the intercalation structure makes the asphalt components form stronger hydrogen bonding connections, which makes the links between different components of the asphalt more stable. The SBS-modified asphalt itself has higher adhesion properties, and the lamellar structure of MMT reinforces the pre-existing SBS stable system, thus increasing the work of cohesion [37]. The energy required to expand the crack at the interface between asphalt and aggregate is called the adhesion work. The energy needed to be absorbed when the asphalt and the mineral were peeled away from each other in the dry state was the adhesion work of the asphalt–aggregate composite. The greater the adhesion work, the more energy needed to be absorbed when the composite was destroyed; therefore, the system was in a more stable state, and the adhesion was better. The adhesion work was calculated as in Equation (10).

(10) $${\Delta G}_{\mathrm{adh},\mathrm{dry}}=\gamma_{\mathrm{binder}}+\gamma_{\mathrm{aggregate}}-\gamma_{\mathrm{binder}-\mathrm{aggreate}}$$

where W_as_ is the adhesive work between asphalt and mineral material, mJ/m^2^; $\gamma_{a}$ is the surface free energy of asphalt, mJ/m^2^; $\gamma_{s}$ is the surface free energy of the mineral material, mJ/m^2^; and ${\;\gamma }_{\mathrm{as}}$ is the interfacial free energy of the asphalt-mineral system, mJ/m^2^.

Substitute Equation (6) into Equation (10) to obtain the adhesive work of asphalt and minerals, as in Equation (11).

(11) $${\Delta G}_{\mathrm{adh},\mathrm{dry}}=2\sqrt{\gamma_{\mathrm{binder}}^{d}\gamma_{\mathrm{aggregate}}^{d}}+2\sqrt{\gamma_{\mathrm{binder}}^{p}\gamma_{\mathrm{aggregate}}^{p}}$$

The adhesion of different asphalts to aggregates is shown in Table 7. From the calculation results, it can be seen that the SBS asphalt binder has a greater adhesion work with the aggregate. With the increase of MMT admixture, the adhesion work also grows, in which the growth rate of base asphalt is better than that of SBS asphalt binder. The degree of bonding between the aggregate and asphalt binder was ranked as sandstone > limestone > basalt, which may be due to the stronger reaction between the more alkaline aggregate and the asphalt presenting acidity, leading to an overall increase in adhesion. The higher the work of adhesion between the binder and the aggregate, the stronger the adhesion between the asphalt and the aggregate, and the greater the work required to strip them both. MMT can increase the adhesion work of base asphalt and SBS asphalt binder up to 19.7% and 8.9%, respectively. The organic modification of the montmorillonite changed from hydrophilic to lipophilic properties, meaning the asphalt in the lamellae existed in a more stable form [38], and therefore the adhesion work of the binder was significantly increased.

### 3.3. Chemical Composition Analysis

#### 3.3.1. Characteristic Functional Group

**Figure 5:**
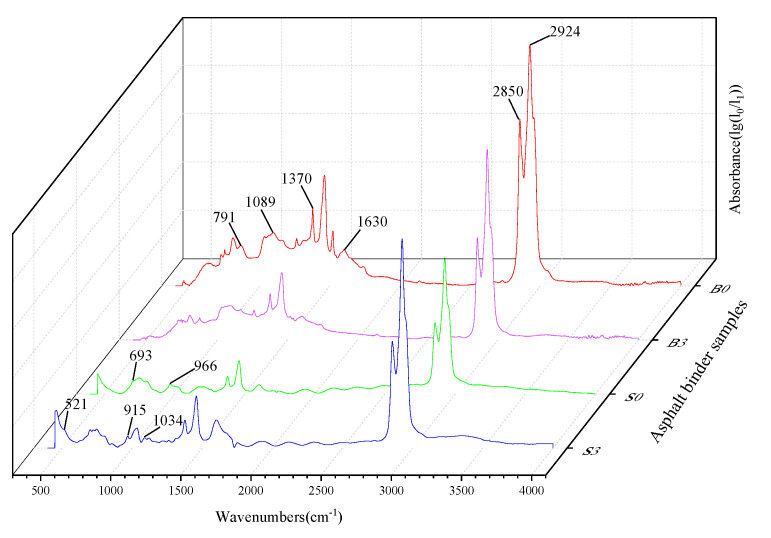
Fourier transform infrared (FTIR) test results of asphalt binder.

Different organic compounds have their own characteristic functional groups, which are formed by specific functional groups and their corresponding infrared absorption bands. The molecular structure characteristics are reflected in the peak positions and intensities in the spectrogram, which identify the chemical groups present in the substance as well as the structural composition. For blended substances, if they are compatible with each other or if there are strong chemical interactions, the spectra may show significant changes in the number, width, or intensity of the characteristic peaks, or the appearance or disappearance of characteristic peaks. The results of the FTIR of the base asphalt, the SBS modified asphalt binder, and the binder with 3% MMT are shown in Figure 5.

In the base asphalt, the absorption peaks at 2924 cm^−1^ and 2850 cm^−1^ are characteristic peaks for alkane C-H asymmetric and symmetric vibrations, respectively. The absorption peak at 1630 cm^−1^ characterizes the conjugated double bond C=C stretching vibration. Notably, 1370 cm^−1^ is a characteristic peak for aliphatic C-H in-plane bending vibration, while 1089 cm^−1^ represents the C-O stretching vibration. The characteristic peak of 791 cm^−1^ represents the olefin C-H out-of-plane bending vibration. For the SBS asphalt binder, there are some differences between the modified asphalt and the base asphalt in the fingerprint region located in the 650–1100 cm^−1^ region. The absorption intensities of the SBS asphalt binder are greater at 690–710 cm^−1^ and 950–980 cm^−1^, and absorption peaks appear at 966 cm^−1^ and 693 cm^−1^, which represent the out-of-plane C-H wobble vibrations on benzene rings and polybutadiene, respectively.

The addition of MMT to the matrix asphalt and SBS asphalt binder resulted in a change in some of the characteristic peaks. The new absorption peaks due to the Si-O-Al bending vibration within the MMT molecule at the position of 521 cm^−1^. Because MMT was organically treated and had good dispersion and compatibility with the matrix asphalt, the binder showed strong adsorption of MMT. Due to the bending vibration of the Al-OH-Al bonding of MMT produces a change in the characteristic peak at the position 915 cm^−1^. The organicization treatment widens the layer spacing and the symmetry of Si-O tetrahedra decreases leading to the enhanced absorption of Si-O and Si-O-Si bonds, thus generating a new absorption peak at 1034 cm^−1^ position due to the Si-O-Si stretching vibration in the crystal structure of MMT.

#### 3.3.2. Molecular Weight Distribution

Substances with different molecular weights of asphalt binder diffused at different rates in GPC machine chromatographic columns, and molecules of different sizes were sequentially separated to obtain corresponding test results [39,40]. The molecular weight distribution of each group of samples was obtained and the SMS, MMS, and LMS of the bound material were calculated according to the elution time and molecular weight conversion. In addition, the weight-average molecular weight (Mw), number-average molecular weight (Mn), and dispersion (D) of the asphalt were also calculated as shown in Table 8. As can be seen, the LMS and MMS percentages of SBS asphalt binder were greater than that of BA, while the SMS content was less than that of BA. With the increase in the amount of MMT modifier, LMS continued to increase, the MMS content of the basic remain unchanged, SMS content had different trends in the decrease, and MMT for BA molecular weight changes had a dramatically impact. Among them, Mw was more sensitive to changes in large molecules in asphalt, Mn was more sensitive to changes in small molecules in asphalt, and D can represent the range of molecular weight distribution in asphalt. The Mw of the SBS asphalt binder was significantly greater than that of BA, which was mainly due to the presence of the polymer. Moreover, the addition of MMT increased the value of Mw, which indicated that the intercalation structure of MMT blocked the flow and diffusion of the lighter components of the binder. The value of Mn of the SBS asphalt binder was small, which also indicated that the content of its light-weight components was less than that of BA. Moreover, the dispersion of the added MMT binder increased, which implied that the content of the various components of MMT modified asphalt was more homogeneous, and the molecular weight distribution was wider.

## 4. Conclusions

The effect of MMT on the performance of base asphalt and SBS asphalt binder was investigated from the perspective of rheological properties, surface energy theory, and chemical properties with the following main findings:

1. With the increase of MMT dosing, the value of elastic recovery of asphalt binder obtained a gradual increase, indicating that the interlayer structure formed by the montmorillonite layer and the asphalt binder plays a coordinating role in the force and deformation of the binder structure. However, the value of irrecoverable creep flexibility decreases with the increase of dosing, indicating that it can improve the high-temperature resistance to plastic deformation of asphalt, which in turn improves the high-temperature performance of the asphalt binder.
2. MMT enhances the surface free energy of the asphalt binder–aggregate system, which mainly increases the component of dispersion fraction. The lamellar structure enhances the interaction between polymer chains and clay layers; therefore, its cohesive power on both base asphalt and SBS asphalt is significantly enhanced. In addition, the experimental results demonstrated the best adhesion ability between the MMT-doped asphalt binder and sandstone.
3. The analysis of FTIR results showed that the interaction between MMT and asphalt is physical, and there is no chemical reaction. There is a good dispersion and compatibility between them because the asphalt shows a strong adsorption to the organic MMT. Absorption peaks generated by stretching or bending vibrations in the crystal structure of MMT can be observed.
4. The chemical composition of the asphalt binder is influenced by the amount of MMT, which increases the LMS content while decreasing the SMS content in the asphalt. The laminar structure restricts the movement of small molecules in the binder and causes the large molecular components to aggregate, enhancing the overall intermolecular forces, which in turn improves the stability of the cross-linked structure in the SBS asphalt binder.
5. It has been demonstrated that MMT can enhance the high-temperature physical properties of asphalt binders and its application in hot areas can help to improve the service quality of pavements and extend the service life of roads. However, the question of whether the application of nanoscale materials to asphalt pavements has any health effects on people needs to be further investigated.

According to the above experimental investigation and discussion, MMT was added to the asphalt to form the interlayer structure, especially in the SBS asphalt binder, and the SBS molecular structure dispersed in the asphalt formed a physical connection system with the laminar structure of MMT. This is reflected in the change in molecular composition and characteristic functional groups, as well as the increase in the overall energy of the asphalt binder, which in turn shows the macroscopic increase in the overall elastic recovery of the asphalt binder under high-temperature conditions.

## Figures and Tables

**Table 1:** Basic properties of asphalt binders.

Technical Parameters	Values		Methods
	Base Asphalt Binder	SBS Asphalt Binder	
Penetration (25 °C, 100 g, 5 s) (0.1 mm)	88.6	72.5	JTG E20-2011 T0604
Ductility (5 °C, 5 cm/min) (mm)	9.5	28.0	JTG E20-2011 T0605
Softening point (°C)	49.0	66.5	JTG E20-2011 T0606
Viscosity (135 °C) (Pa·s)	0.58	1.62	JTG E20-2011 T0625

**Table 2:** Physical properties of MMT.

Physical Properties	MMT
MMT content (%)	96–98%
Apparent density (g cm^−3^)	0.25–0.40
Water content (%)	<5%
Radius-thickness ratio	200
Average wafer thickness (nm)	15

**Table 3:** Chemical compositions of aggregate.

Main Chemical Composition	Limestone	Sandstone	Granite
SiO_2_ (%)	4.2	34.72	67.4
Al_2_O_3_ (%)	2.8	5.39	16.8
Fe_2_O_3_ (%)	0.6	2.67	1.6
MgO (%)	1.2	6.69	2.1
CaO (%)	54.5	16.15	3.2

**Table 4:** Name of asphalt binder samples.

Name of Samples	Composition
B0	Base asphalt
B1	Base asphalt with 1% MMT
B3	Base asphalt with 3% MMT
B5	Base asphalt with 5% MMT
S0	SBS modified asphalt
S1	SBS modified asphalt with 1% MMT
S3	SBS modified asphalt with 3% MMT
S5	SBS modified asphalt with 5% MMT

**Table 5:** Test results of surface free energy of aggregate and measured liquid.

Sample Type		Surface Free Energy Components (mJ/m^2^)		
		$\gamma^{d}$	$\gamma^{p}$	γ
Detection liquid	Deionized Water	20.6	52.2	72.8
	Formamide	38.9	19.6	58.5
	Glycerol	34.0	30.0	64.0
Aggregate	Limestone	26.3	13.9	40.2
	Sandstone	27.4	18.1	45.5
	Basalt	23.7	16.1	39.8

**Table 6:** Rheological indicators test results and coefficient of variation.

Binder Type	Final Strain		R_0.1_		R_3.2_		J_0.1_		J_3.2_	
	Avg.	CV	Avg.	CV	Avg.	CV	Avg.	CV	Avg.	CV
B0	11,670.12	0.73	4.81	0.13	1.21	0.19	0.45	0.34	0.54	0.36
B1	9233.18	0.47	5.32	0.36	1.78	0.21	0.39	0.36	0.42	0.21
B3	5549.31	0.28	7.78	0.28	4.61	0.14	0.31	0.28	0.41	0.12
B5	4786.26	0.36	12.33	0.58	8.14	0.61	0.26	0.18	0.34	0.19
S0	5426.15	0.67	21.84	0.25	12.40	0.33	0.15	0.25	0.18	0.24
S1	4359.28	0.22	24.57	0.47	15.23	0.28	0.12	0.13	0.16	0.38
S3	2542.56	0.49	34.26	0.33	20.76	0.29	0.08	0.21	0.10	0.27
S5	2159.57	0.12	36.13	0.55	21.27	0.39	0.05	0.19	0.08	0.19

Avg. (%) and CV (%) refer to the Average Value and the Coefficient Variation, respectively.

**Table 7:** Test results of work of cohesion of asphalt binders and work of adhesion to aggregate.

Binder Type	$\gamma_{binder}^{d}$ (mJ/m^2^)	$\gamma_{binder}^{p}$ (mJ/m^2^)	$\gamma_{binder}$ (mJ/m^2^)	$\Delta G_{coh}$ (mJ/m^2^)	$\Delta G_{adh,dry}$ (mJ/m^2^)		
					Limestone	Sandstone	Basalt
B0	9.2	2.05	11.25	22.5	41.82	43.97	41.04
B1	10	1.89	11.89	23.78	42.72	44.84	41.85
B3	13.25	1.91	15.16	30.32	47.68	49.91	46.56
B5	15.58	1.75	17.33	34.66	50.39	52.62	49.08
S0	15.79	1.29	17.08	34.16	49.26	51.31	47.84
S1	16.58	1.31	17.89	35.78	50.33	52.41	48.87
S3	18.44	1.20	19.64	39.28	52.25	54.32	50.65
S5	19.27	1.35	20.62	41.24	53.72	55.89	52.11

**Table 8:** Test results of gel chromatography.

Binder Type	LMS (%)	MMS (%)	SMS (%)	Mw	Mn	D
B0	14.4	57.1	28.5	3879	1610	2.41
B1	15.1	57.6	27.3	3895	1617	2.41
B3	16.6	56.9	26.5	3946	1617	2.44
B5	17.9	57.2	24.9	4018	1620	2.48
S0	19.2	59.3	21.5	4165	1698	2.45
S1	20.8	58.2	21.0	4212	1712	2.46
S3	23.8	56.4	19.8	4288	1736	2.47
S5	25.2	56.6	18.2	4351	1740	2.50

## Abbreviations

γ	Surface free energy
$\gamma^{d}$	Dispersion component of surface energy
$\gamma^{p}$	Polar component of surface energy
$\gamma_{\mathrm{SL}}$	Surface energy at the solid-liquid interface
$\gamma_{S}$	Surface energy of the solid
$\gamma_{L}$	Surface free energy of the liquid
θ	Contact angle
${\Delta G}_{\mathrm{coh}}$	Cohesive work of the asphalt binder
${\Delta G}_{\mathrm{adh},\mathrm{dry}}$	Adhesive work between asphalt and mineral material in dry condition
R	Deformation recovery percentage
R_diff_	Creep recovery rate difference ratio
$\varepsilon_{p}$	Peak strain in each loading cycle
$\varepsilon_{r}$	Residual strain in each loading cycle
$\varepsilon_{0}$	Initial strain in each loading cycle
J_nr_	Nonrecoverable creep compliance
J_diff_	Difference ratio of non-recoverable creep compliance
σ	Applied stress level
LMS	Large molecular size
MMS	Medium molecular size
SMS	Small molecular size
Mw	Weight-average molecular weight
Mn	Number-average molecular weight
D	Dispersion
